# Long-Term Survival for Traumatic Spinal Cord Injury in British Columbia, Canada: A Retrospective Evaluation of 20 Years of Linked Health Care Data

**DOI:** 10.1089/neur.2025.0057

**Published:** 2025-06-16

**Authors:** Michael Bond, Aidan Beresford, Vanessa Noonan, Naama Rotem-Kohavi, Marcel Dvorak, Brian Kwon, Guiping Liu, Jason Sutherland

**Affiliations:** ^1^ Centre for Health Services and Policy Research, University of British Columbia, Vancouver, Canada.; ^2^ Praxis Spinal Cord Institute, Vancouver, Canada.; ^3^ International Collaboration on Repair Discoveries (ICORD), University of British Columbia, Vancouver, Canada.; ^4^ Combined Neurosurgery and Orthopaedic Spine Program, University of British Columbia, Vancouver, Canada.

**Keywords:** administrative data, epidemiology, mortality, surgery, trauma, traumatic spinal cord injury

## Abstract

Patients living with traumatic spinal cord injury (TSCI) have seen many improvements in care and treatment, but life expectancy still falls below the general population. Measuring long-term survival rates and characterizing causes of death are required to identify ways of improving well-being and reduce premature mortality. The study conducted a retrospective analysis of population-based administrative and clinical data from 2001 to 2021 to measure long-term survival of TSCI, mortality predictors, and cause of death. Population-based hospital records linked with administrative databases in British Columbia, Canada, were used to identify those with TSCIs. Demographic and clinical summary statistics were calculated. Mortality rates for 1-, 5-, 10-, 15-, and >15-year survival were calculated using Kaplan–Meier methods. Factors associated with mortality throughout the study period were identified with Cox models. During the study period, 3624 patients were identified with TSCI. The mean age was 51.1 years (SD 21.19) and 2718 (75.0%) were male. Mortality rates at 1, 5, 10, 15, and >15 years were 11.2%, 19.6%, 25.4%, 28.3%, and 29.1%, respectively. Factors associated with mortality included cervical spine injuries, more comorbidities, older age, lower household income, presence of traumatic brain injury, and greater severity of initial injury (*p* < 0.001). Cardiac disease (22.3%) was the most common cause of death in TSCI patients followed by respiratory diseases (10.2%) and neoplasms (8.5%). The long-term survival of TSCI patients is a significant concern, and preventative measures to avoid injury are critical. Among those suffering TSCI, particularly high death rates are observed in those with cervical injuries, multiple comorbidities, and advanced age. Interventions are needed to reduce premature death among TSCI patients compared with the population.

## Introduction

Traumatic spinal cord injury (TSCI) is a devastating condition that causes physical disability due to loss of motor and sensory function.^1,2^ TSCIs are the result of injuries seen among younger populations due to high-energy mechanisms such as motor vehicle collisions and increasingly among elderly populations from low-energy falls^3,4^ TSCI is a relatively rare injury, with global estimates of 23 per million persons, and Canadian estimates of between 30 and 50 per million.^5^ The complex nature of TSCI requires acute management at large centers and the mobilization of health care resources for initial treatment.^6–8^ Depending on the severity of the injury, there are many variations of initial presentations, expected recovery trajectories, and rehabilitation potential for patients requiring health care systems that can adapt to these challenges and change their focus across patients’ life course^9–11^

Significant changes in the treatment of TSCI have occurred over the last several decades, including improved trauma care, advanced surgical, medical, and rehabilitative care, and the development of specialized spinal injury treatment centers.^9,12^ As a result, the management of TSCI in the acute setting has led to improved short-term survival and fewer in-hospital complications.^13,14^ However, even with ideal medical attention and appropriate rehabilitation care, the life expectancy for patients living with a TSCI falls below the general population; moreover, long-term survival has reportedly not changed significantly according to older reports.^15,16^

Based on current information, between 12% and 45% of TSCI patients die within the first year after their TSCI.^15,17,18^ Long-term data have demonstrated that 10-year mortality rates are between 10% and 15%.^19,20^ Risk factors for premature mortality among TSCI patients have associated elevated mortality rate with level of spinal cord injury, severity of neurological injury, ventilator dependence, and age.^15,21–24^ However, there is a paucity of data on the mortality rates among patients with TSCI as most available literature measures mortality during acute hospitalization associated with the injury. Understanding of the survival of patients with TSCI can greatly aid in health care system and rehabilitation planning for this complex population.

With the availability of improving population registries and administrative and clinical data, there are new opportunities for generating insights into long-term mortality rates among TSCI patients that can guide health services planning and policies impacting patients with TSCI across their life course. This study uses unique sources of linked data to measure the mortality rates of individuals with TSCI in the province of British Columbia (BC). Furthermore, this study identifies risk factors at the time of injury associated with premature mortality and the subsequent cause of mortality for individuals with TSCI.

## Methods

### Study population and data

This study was based on population-based retrospective observational data. A cohort was created that included all patients treated for acute cervical and thoracic spinal cord injury in acute care hospitals between 2001 and 2021. Patients who had lumbar and sacral nerve root injuries (including cauda equina syndrome) were also included as they also experience mobility loss, complex medical issues, and bowel/bladder dysfunction and were felt to also represent the intended patient population. Those spinal cord injuries that were not deemed to be traumatic were excluded, such as iatrogenic and oncologic diagnoses. Ethics approval was obtained from the University of BC Research Ethics Board (REB#: H22-02696).

Individuals with TSCI were identified through population-based analyses of data from the repositories of Population Data BC, which is an organization that amalgamates data for research purposes from several different sources of population-level administrative datasets. This study utilized linked population-based data from hospital discharge records, physician billing data, and provincial demographic statistics information.^25^ Population Data BC facilitates linkage among the data to provide patient information, including longitudinal health through a deidentified process. For this study, TSCI patients were linked across several sources of data through Population Data BC, including the Canadian Institute for Health Information’s Discharge Abstract Database (DAD), BC’s Provincial Vital Statistics Agency (deaths), the BC Trauma Registry, and the Rick Hansen Spinal Cord Injury Registry (RHSCIR).^26–32^ The protocol for linkage has been described elsewhere.^33^

Patients were identified for inclusion in the cohort using the *International Classification of Disease* (ICD) codes in the DAD. The codes and mapping were validated elsewhere and included all levels and severity of spinal cord injury, including cervical, thoracic, lumbar complete/incomplete injuries, and cauda equina syndrome (see Supplementary Appendix).^34^

### Clinical and demographic variables

Variables included in the analyses were age (categorized as <35, 35 to 64, >65), sex, Charlson comorbidity index,^35^ urban or rural place of residence at the time of injury, surgical treatment, and length of index hospital admission. Clinical variables included date of admission, date of injury, mechanism of injury, spinal column level (cervical, thoracic, lumbar, sacral/cauda equina), injury severity score (ISS) (categorized as <25 or ≥25),^36^ neurological injury (complete vs. incomplete), functional impairment (paraplegia vs. tetraplegia), presence of traumatic brain injury (TBI), and mechanism of injury (motor vehicle collision, falls, or other). Socioeconomic status at the time of injury was determined by using the Quintile of Adjusted Income per Person Equivalent (QAIPPE), which is a measure of neighborhood income per person equivalent, adjusted for household size, and is based on census summary data from Statistics Canada.^37^ All variables were extracted from the time of admission for the initial traumatic injury occurred.

### Study outcome

The primary outcome of this study was death, identified through the Vital Statistics Agency.^28^

### Statistical analyses

Demographic and clinical variables were analyzed using simple summary statistics, means (standard deviations) for continuous variables and counts (percentages) for categorical variables were reported. Mortality rates were assessed from the time of injury. The 1-, 5-, 10-, 15-, and >15-year mortality rates were calculated using Kaplan–Meier functions and life tables. Rates were stratified by sex, level of injury, and neurological status to identify differences. Log-rank tests were used to compare survival rates between stratified groups.

To evaluate risk factors associated with mortality, a Cox-proportional hazards model was used. Variables included in the model were age, sex, socioeconomic status, year of injury, surgical treatment, injury level, complete or incomplete cord injury, ISS, Charlson comorbidity index, TBI, and urban and rural. The assumptions of the Cox proportional hazard model were evaluated before performing the regression, and model fit was evaluated using the AIC and evaluation of the residuals. All statistical analyses were performed using SAS v.9.4.

## Results

Over the 21-year study period, 3624 patients were included in the cohort. The mean age was 51.1 years (SD 21.19) and 2718 (75.0%) were male. Surgery rates were found to be 48.5% during hospitalization for the injury. Patients were hospitalized in acute care for an average of 67.4 days (SD 98.00) (see Table 1).

**Table 1.** Demographic and Clinical Data for Patients with Traumatic Spinal Cord Injury from 2001 to 2021

Variable	Value
*N*	3624
Age (SD)	51.1 (21.19)
Male	2718 (75.0%)
Charlson Comorbidity Index	
0	2977 (82.1%)
1	396 (10.9%)
2	154 (4.2%)
≥3	97 (2.7%)
Quintile of annual income per person equivalent	
1	863 (23.8%)
2	727 (20.1%)
3	687 (19.0%)
4	641 (17.7%)
5	620 (17.1%)
6 NA or missing	86 (2.4%)
Injury level	
Cervical (C1–C7)	2520 (69.5%)
Thoracic (T1–T12)	647 (17.9%)
Lumbar (L1–L5)	375 (10.3%)
Sacral/cauda (S1–S5)	82 (2.3%)
Neurological severity	
Complete injury	729 (20.1%)
Incomplete injury	2786 (76.9%)
Tetraplegia	2520 (69.5%)
Surgery	
Yes	1757 (48.5%)
No	1867 (51.5%)
Injury severity score (ISS) (SD)	24.3 (13.95)
Region	
Rural	573 (15.8%)
Urban	2982 (82.3%)
Unknown	69 (1.9%)
Cause of injury	
Falls	1809 (49.9%)
Transport	1324 (36.5%)
Other	491 (13.5%)
LOS (acute) in days (SD)	67.4 (107.52)

LOS, length of stay.

**FIG. 1.**
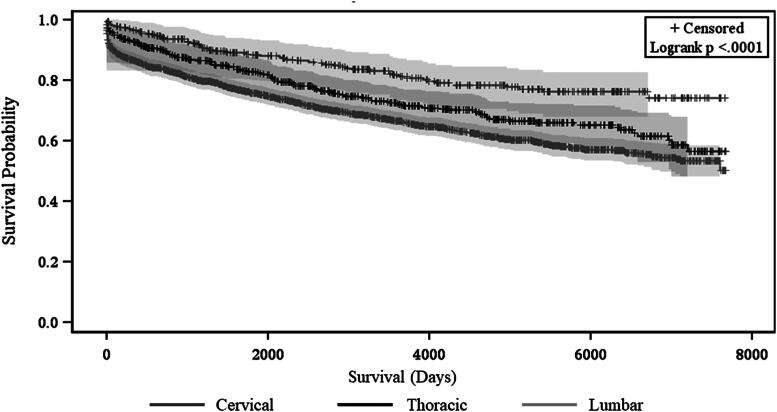
Kaplan–Meier survival curve stratified by injury level (cervical, thoracic, and lumbar).

**FIG. 2.**
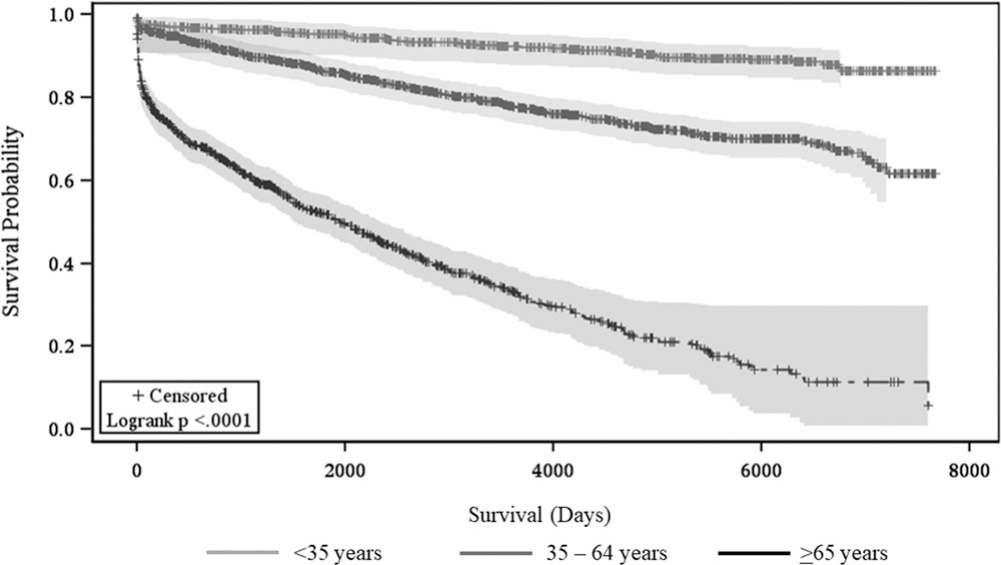
Kaplan–Meier survival curve of traumatic spinal cord injury (TSCI) stratified by age (<34, 35–64, >65).

There were 1054 patients in the cohort who died (29.1%). The cumulative mortality rate since the time of injury was 11.2% at 1 year, 19.6% at 5 years, 25.4% at 10 years, 28.3% at 15 years, and 29.1% for over 15 years (see Table 2). Kaplan–Meier survival analyses found that cervical spine injuries were more likely to have early mortality compared with those with thoracic, lumbar, or sacral injuries (log-rank test, *p* < 0.001, Fig. 1). Those who were older than 65 years had the shortest survival compared with those in both the 35–64-year-old and <35-year-old bracket (log-rank test, *p* < 0.001, Fig. 2). Sex was not associated with differences in survival (log-rank test, *p* > 0.05).

**Table 2.** Cumulative Mortality Percentages Out of Hospital for Identified Traumatic Spinal Cord Injury Patients from 2001 to 2021

Years since the first admission	Cumulative deaths	Number of survivals	Survival (%)	Deaths (%)
0	0	3624	100	0
1	406	3218	88.8	11.2
5	710	2914	80.4	19.6
10	921	2703	74.6	25.4
15	1024	2600	71.7	28.3
>15	1054	2570	70.9	29.1

Cox-proportional hazard regression found that cervical spine injuries, older age, greater number of medical comorbidities, concurrent TBI, higher ISS (>25), and those who did not have surgery had shorter survival (*p* < 0.001). Furthermore, cohort members in the lowest two socioeconomic brackets (as measured by QAIPPE) had shorter survival compared with the highest income bracket (see Table 3).

**Table 3.** Cox Proportional Hazard Model for Traumatic Spinal Cord Injury Patients Including Hazard Ratios and Confidence Intervals for Demographic and Clinical Variables Associated with Mortality Out of Hospital

Variable	HR	95% CI	*p* Value	
Age				
<35	Ref.			
35–64	3.26	2.54–4.19	**<0.001**	
+65	14.76	11.43–19.08	**<0.001**	
Sex				
Male	1.16	1.01–1.34	**0.036**	
Female	Ref.			
Injury level				
Lumbar (L1–L5)	Ref.			
Thoracic (T1–T12)	1.03	0.77–1.39	0.820	
Cervical (C1–C7)	1.35	1.04–1.75	**0.023**	
Other/sacral	1.11	0.61–2.04	0.724	
Charlson Comorbidity Index				
0	Ref.			
1	1.58	1.34–1.86	**<0.001**	
2	2.38	1.91–2.96	**<0.001**	
≥3	3.28	2.52–4.27	**<0.001**	
Region				
Rural	0.87	0.73–1.04	0.134	
Urban				
Neurological severity				
Incomplete	Ref.			
Complete	1.70	1.42–2.04	**<0.001**	
Injury severity score				
<25	Ref.			
≥25	1.67	1.39–2.02	**<0.001**	
TBI				
Absent	Ref.			
Present	1.34	1.13–1.58	**<0.001**	
Spine surgery (Yes/No)				
Yes	Ref.			
No	1.68	1.45–1.94	**<0.001**	
Socioeconomic status (QAIPPE)				
1 Lowest	1.44	1.19–1.76	**<0.001**	
2	1.32	1.07–1.63	**0.010**	
3	1.15	0.92–1.42	0.219	
4	1.19	0.95–1.48	0.128	
5 Highest	Ref.			
Acute length of hospital stay (LOS)	1.00	1.00	0.382	
Specialized versus nonspecialized care				
Specialized	1.03	0.89–1.18	0.692	
Nonspecialized care	Ref.			

CI, confidence interval; HR, hazard ratio; TBI, traumatic brain injury.

Significance set at *p* < 0.05 is in bold text.

Throughout the study it was observed that cardiac disease (22.3%) was the most frequent cause of death in the TSCI patient cohort, followed by respiratory diseases (10.2%), neoplasms (8.5%), endocrine-related disease (5.4%), nervous system-related diseases (4.7%), mental-related disease (3.9%), renal-related disease (3.7%), accidents (3.5%), musculoskeletal disease (1.3%), suicide (1.0%), and others (35.8%) (see Table 4).

**Table 4.** Cause of Death for Patients with Traumatic Spinal Cord Injury from 2001 to 2021

Cause of death	*N*	Deaths (%)
Cardiac	157	22.3
Respiratory	72	10.2
Neoplasms	60	8.5
Endocrine-related disease	38	5.4
Nervous system-related disease	33	4.7
Mental-related disease	28	3.9
Renal-related disease	26	3.7
Accidents	25	3.5
MSK-related disease	9	1.3
Suicide	7	1.0
Other (all other causes)	254	35.8

## Discussion

This study analyzed population-level data over a 20-year study period to evaluate the mortality rates and risk factors for long-term mortality in patients who experienced a TSCI in the province of BC, Canada. Results demonstrated that the 1-year mortality rate among those with TSCI after their initial injury was 11.2%, 19.6% at 5 years, 25.4% at 10 years, 28.3% at 15 years, and 29.1% at more than 15 years. Risk for earlier mortality was found to be associated with older age, cervical cord injuries, greater number of pre-existing medical comorbidities, TBI, higher severity of initial injury, and those who did not have surgery at the time of injury. Identifying those with TSCI who have a higher likelihood of early mortality can inform clinical decisions and surveillance.

The findings of this study are consistent with existing literature that has evaluated 1-year long-term survival for TSCI. In the United States, 1-year mortality rates are 16.2%, with similar findings in Australia at 12.3%, compared with this study’s finding of 11.5%.^15,17^ Our study reports a higher 10-year mortality when compared with past literature. A systematic review of the international 10-year mortality rates of spinal cord injury demonstrated a range of 7–19% globally.^38^ In Canada, mortality rates have been shown in older studies looking at younger age groups, showing a much lower mortality rate of 8% in the first 10 years after injury.^39^ The current study represents an update on 10-year mortality rates, given previous studies demonstrating a significant shift in the number of patients affected by TSCI who are geriatric. Thus, this higher mortality rate likely reflects the changes seen from both updated data and this shifting demographic. Population-based linked datasets are useful for studying uncommon and rare conditions such as TSCI.^40^ The results from this analysis help to better understand the rates of mortality for patients with TSCI in BC, including those that may not be captured in large academic center registries using population-level data. It also helps to identify factors associated with early mortality, and to the best of our knowledge is the one of the only studies evaluating long-term follow-up and mortality rates in Canada for more than 10 years.

With the demographics of TSCI changing as populations age, more elderly patients are being treated for TSCI; this study found that those who were older than 65 years had higher mortality rates at all time points during a 15-year follow-up.^41^ Fallah et al. (2022) have also found through machine learning that older age is a predictor for early mortality and were able to develop risk models for mortality.^42^ The treatment of TSCI in the geriatric population can be complex, given that they often have higher medical comorbidities, greater frailty, and may not be candidates for certain interventions such as surgical management.^43–45^ Furthermore, elderly patients are more likely to experience complications related to their TSCI, such as respiratory illness, pressure injuries, and cardiovascular events, all of which can lead to earlier mortality.^46–48^ The surgical rate in this study was 48.5%, which although low may reflect the increase in elderly patients as it has been found that often those older than 65 are less likely to undergo surgical management compared with younger patients.^49,50^ Finally, participation in rehabilitation after TSCI is an important component of functional recovery to prevent complications and improve outcomes; however, geriatric patients often are not deemed appropriate candidates for comprehensive TSCI rehabilitation due to medical issues and lack of participation.^51^ There is a pressing need for the development of clear clinical pathways and multidisciplinary rehabilitation programs with geriatric specialists for those who experience TSCI at an older age to improve treatment and long-term outcomes and mortality.

Patients in this study who had a concurrent TBI, cervical spine injury, and a higher trauma severity score (ISS) at the time of their TSCI were associated with higher mortality risk. It is known that those who have more complex TSCI cases are more likely to require additional rehabilitation, have more complex needs throughout the life course, and are more likely to experience early mortality.^42,52,53^ Those with cervical spine injuries are faced with worse neurological injuries that can cause secondary medical issues such as impaired cardiovascular and autonomic function, potential ventilation requirements, and poor mobility leading to a lower overall health status.^54,55^ Patients with concurrent TBI specifically can have neurological and cognitive sequelae from their injury that make them less likely to engage in rehabilitation, putting them at higher risk of having seizure disorders, gastrointestinal motility issues, and mental health conditions that affect the overall quality of life.^56,57^ As TBIs are more likely to occur in patients with concurrent additional severe injuries, an important aspect of treating these complex injuries is to identify teams or centers that are equipped to manage complex trauma. Recent evidence has suggested that improving mortality in patients with a high ISS can be achievable using specialized trauma centers and systems.^58,59^ However, these studies traditionally have evaluated in-hospital or 1-year mortality, and further research is needed to evaluate the outcome of specialized centers for TSCI in long-term follow-up.

In the last several decades, there have been significant improvements in surgical techniques, understanding the underlying mechanism of TSCI, and the development of more intense rehabilitation methods, all of which have combined to lead to improvements in initial injury mortality rates.^60^ Despite these efforts and advances, it is not apparent that there has been a significant improvement in the overall life expectancy for those who suffer a TSCI.^16^ Community-based care pathways that follow patients long term and reduce potential preventable medical complications are not implemented at a systems level, and thus, only a patchwork of services and care are available to patients with TSCI.^61^

The causes of death after TSCI have been well studied, and in a large 70-year cohort follow-up in the United Kingdom it was found that of those who died, 29.3% of patients died from respiratory complications, 26.7% from cardiovascular, 13.9% from cancers, 11.9% urogenital, 5.3% digestive, and 4.5% from suicide.^62^ In another large study, conducted in Germany over a 21-year period, the main causes of death in this cohort were similar to cardiovascular disease, pneumonia, and complications from pressure sores.^63^ Our study identified that the leading cause of death in patients is cardiac disease and respiratory disease, which is consistent with existing literature.

In Canada, which is the setting of this population-based study, there is a significant gap in the care of patients transitioning between hospital-based care and community care, with limited care pathways or care coordinators to facilitate this crucial juncture.^64,65^ Out-of-pocket costs, such as mobility aids, medications, home care, and rehabilitation, often cause significant expenses to the individual, and in many countries, including Canada, these services are only partially insured by public health insurance programs. To add to this disparity, there are also regional variations in accessing care since many specialty services are only available in urban settings.^64^

This study demonstrated that those in lower income brackets were more likely to have earlier mortality compared with those in the highest income bracket, further emphasizing the potential link between accessing care and improving outcomes. This finding is consistent with other research recognizing disparities in survival for those of lower socioeconomic groups.^66^ Mechanisms need to be established for additional coverage for appropriate health services or technologies for patients with TSCI to ensure that they have equitable access to the care and services they require for rehabilitation, medical care, and access to specialty services.

There are limitations to this study. This was a study drawing data from only one setting or province in Canada. Thus, the results may not be representative of other jurisdictions’ health care systems for TSCI survivors. However, it is an evaluation of a large sample of patients across a large geographic area and adds to the existing literature by providing over 15 years of mortality data. Second, many rehabilitation services and community-based home care are not covered through the provincial insurance system and are unfortunately not identified or measured to provide additional context to this study’s findings. Finally, the cohort was created based on hospital administrative data’s diagnostic codes. These codes are entered generally through hospital record review but may be inaccurately entered and thus may have errors and inaccuracies in reporting; however, given that the care for spinal cord injury often involves a complex stay in hospital, the diagnosis is often complete for these patients and the validation of the cohort creation for this study has been previously assessed.^33^

## Conclusion

This study reported 1-, 5-, 10-, 15-, and >15-year mortality rates of TSCI in a population with a range of characteristics and identified risk factors for mortality. The results provide insight into health care providers’ services and help measure predictors of early mortality. To address potentially modifiable factors for premature mortality, access to services that can prevent potential complications and enhance rehabilitation is needed. Further research is needed to both implement and evaluate the effectiveness of care pathways in the community for patients with TSCI in improving long-term mortality rates.

## Transparency, Rigor, and Reproducibility Statement

This study utilized discharge administrative data from Population Data BC for analysis. The data were obtained upon request from 2001 to 2021. Data use agreements prohibit sharing. Access to data provided by the Data Stewards is subject to approval but can be requested for research projects through the Data Stewards or their designated service providers. The following datasets were used in this study: Consolidation file (includes demographics, registry, and census geodata), Hospital Separations, Medical Services Plan (MSP), Vital Events and Statistics-Deaths, RHSCIR, BCTR, and Vertebase/QISpine. You can find further information regarding these datasets by visiting the PopData project webpage (https://my.popdata.bc.ca/project_listings/15-119).

All inferences, opinions, and conclusions drawn in this publication are those of the author(s), and do not reflect the opinions or policies of the Data Steward(s).

Statistical analysis was performed on SAS. Sample size and power calculations were not conducted as the dataset obtained was exceptionally large, ensuring the study was adequately powered. Due to the study’s retrospective nature, it was not preregistered nor analyzed. The sample size was determined by the number of patients diagnosed in hospital with TSCI. The study utilized validated ICD-10 code definitions for inclusion. After screening, 3624 patients were included. Investigators knowledgeable in the relevant topic assessed key inclusions and outcomes to report on. Statistical analysis was performed on SAS. Sample size and power calculations were not conducted, as the dataset obtained was exceptionally large, ensuring the study was adequately powered.

## Abbreviations Used

BC: British Columbia
DAD: Discharge Abstract Database
ICD: International Classification of Disease
ISS: Injury Severity Score
LOS: Length of Stay
QAIPPE: Quintile of Adjusted Income per Person Equivalent
RHSCIR: Rick Hansen Spinal Cord Injury Registry
TBI: Traumatic Brain Injury
TSCI: Traumatic Spinal Cord Injury
