# Supplementary material for: Long-Term Survival for Traumatic Spinal Cord Injury in British Columbia, Canada: A Retrospective Evaluation of 20 Years of Linked Health Care Data
Source: Neurotrauma Rep. 2025 Jun 16;6(1):551–9. doi: 10.1089/neur.2025.0057 (PMC12235124; doi:10.1089/neur.2025.0057)
Supplement: Supplementary Appendix [file neur.2025.0057_supplementary_appendix.docx]

Appendix 1: International Classification of Diseases (ICD)-10 diagnosis codes for Traumatic Spinal Cord Injury

| **ICD-10 codes for TSCI** | | |
| --- | --- | --- |
| S14.0 | Concussion and oedema of cervical spinal cord |  |
| S14.10 | Complete lesion of cervical spinal cord |  |
| S14.11 | Central cord lesion of cervical spinal cord |  |
| S14.12 | Anterior cord syndrome of cervical spinal cord |  |
| S14.13 | Posterior cord syndrome of cervical spinal cord |  |
| S14.18 | Other injuries of cervical spinal cord |  |
| S14.19 | Unspecified lesion of cervical spinal cord |  |
| S24.0 | Concussion and oedema of thoracic spinal cord |  |
| S24.10 | Complete lesion of thoracic spinal cord |  |
| S24.11 | Central cord lesion of thoracic spinal cord |  |
| S24.12 | Anterior cord syndrome of thoracic spinal cord |  |
| S24.13 | Posterior cord syndrome of thoracic spinal cord |  |
| S24.18 | Other injuries of thoracic spinal cord |  |
| S24.19 | Unspecified lesion of thoracic spinal cord |  |
| S34.0 | Concussion and oedema of lumbar spinal cord |  |
| S34.10 | Complete lesion of lumbar spinal cord |  |
| S34.11 | Central cord lesion of lumbar spinal cord |  |
| S34.12 | Anterior cord syndrome of lumbar spinal cord |  |
| S34.13 | Posterior cord syndrome of lumbar spinal cord |  |
| S34.18 | Other injuries of lumbar spinal cord |  |
| S34.19 | Unspecified lesion of lumbar spinal cord |  |
| S34.30 | Laceration of cauda equina |  |
| S34.38 | Other and unspecified injury of cauda equina |  |
| T06.0 | Injuries of brain and cranial nerves with injuries of nerves and spinal cord at neck level |  |
| T06.1 | Injuries of nerves and spinal cord involving other multiple body regions |  |
